# SOCIOECONOMIC STATUS AND THE RELATIONSHIP OF PHYSICAL PERFORMANCE WITH ACTIVITIES OF DAILY LIVING

**DOI:** 10.1093/geroni/igae098.2816

**Published:** 2024-12-31

**Authors:** Grace Chang, Rahul Malhotra, Benjamin Seligman

**Affiliations:** University of California Los Angeles, Los Angeles, California, United States; Duke-NUS Medical School, Singapore, Singapore; University of California Los Angeles David Geffen School of Medicine, Los Angeles, California, United States

## Abstract

The translation of reduced intrinsic capacity, assessed through physical performance such as gait speed and grip strength, to diminished functional ability, such as limitations in activities of daily living (ADLs), can be modified by one’s environment. Socioeconomic status (SES), linked with access to resources, may thus modify the relationship between physical performance and ADL limitations. We study this question in a cohort of older adults, using data from the 2016 wave of the Health and Retirement Study. Performance was measured using gait speed and grip strength, adjusted for sex and height/BMI respectively. ADL limitation was assessed using five Katz ADLs: bathing, toileting, eating, dressing, and transferring from bed. We considered three SES measures: highest education level, household wealth, and household income. Analysis was by negative binomial regression of the number of ADL limitations on performance and/or SES with adjustment for age and sex. Individually, each performance and SES measure was associated with ADL limitations. In regressions including grip strength, SES was associated with ADL limitations, and wealth and income had interactions with grip strength. For gait speed, only wealth was associated with ADL limitations, though education had an interaction with gait speed. Our mixed results for effect modification by SES of the association between physical performance and ADL limitations do suggest the role of SES as an effect modifier, contingent on the measures being used for assessing physical performance and SES. However, they also suggest that performance may mediate the relationship between SES and ADL limitations.

